# Molecular epidemiology, drug resistance, and virulence gene analysis of *Streptococcus agalactiae* isolates from dairy goats in backyard farms in China

**DOI:** 10.3389/fcimb.2022.1049167

**Published:** 2023-01-09

**Authors:** Hongfei Shi, Mengxiao Zhou, Zhengtian Zhang, Yun Hu, Shiyang Song, Ruiqing Hui, Long Wang, Guoguang Li, Lunguang Yao

**Affiliations:** ^1^ Henan Provincial Engineering and Technology Center of Animal Disease Diagnosis and Integrated Control, Henan Provincial Engineering Laboratory of Insects Bio-reactor, Nanyang Normal University, Nanyang, China; ^2^ College of Animal Husbandry and Medical Engineering, Nanyang Vocational College of Agriculture, Nanyang, China; ^3^ Animal Husbandry and Fishery Department, Heilongjiang State 853 Farm Limited Company, Shuangyashan, China

**Keywords:** *Streptococcus agalactiae*, mastitis, goat, antimicrobial resistance, virulence gene

## Abstract

*Streptococcus agalactiae* infections may lead to clinical or subclinical mastitis in dairy animals when it invades the mammary gland. In this study, 51 *S. agalactiae* strains were isolated from 305 milk samples that were collected from goats with mastitis in 13 provinces of China. The antimicrobial resistance of *S*. *agalactiae* was determined by disk diffusion methods against 18 antibiotics from six classes. In addition, multilocus sequence typing (MLST), and the presence of resistance and virulence genes was determined by PCR analysis. Seven sequence types in five clonal complexes were identified according to MLST; CC103 and CC67 strains were predominant, with rates of 45.1% and 39.2%, respectively. All isolates (100%) were multiresistant to three or more antimicrobial agents. *S*. *agalactiae* isolates had a 100% resistance rate to penicillin, oxacillin, and amoxicillin, followed by doxycycline (82.4%), tetracycline (76.5%), and amikacin (74.5%). The lowest resistance was observed for ciprofloxacin (29.4%), which varied in five different regions. The detection rates of six classes of antimicrobial-related genes were calculated as follows: 33 (64.7%) for β-lactam-related resistance gene, 12 (23.5%) for tetracyclines, 11 (21.6%) for quinolone-related resistance genes, 10 (19.6%) for aminoglycosides, 7 (13.7%) for macrolides (*ermA*, *ermB*, and *mefA*), and 3 (5.9%) for lincosamide (*lnu(B)*). Regarding virulence genes, profile 1 (*bca cfb*-*cspA*-*cylE-hylB-bibA-pavA-fbsA-fbsB*) was the most prevalent, with a detection rate of 54.9%. This work provides a primary source related to the molecular epidemiology of *S. agalactiae* in dairy goat herds in China and will aid in the clinical treatment, prevention, and control of mastitis.

## Introduction

*Streptococcus agalactiae* (*S*. *agalactiae*) is a species of Gram-positive chain-forming cocci, and is also called group B *Streptococcus*. It mainly affects humans (Lannes-Costa et al., 2021; Nguyen et al., 2021; Tavares et al., 2022), cattle (Keefe, 2012; Reyher et al., 2012; Kabelitz et al., 2021), and fish (Alazab et al., 2022; Piamsomboon et al., 2022; Sapugahawatte et al., 2022). In dairy animals, *S. agalactiae* invades the mammary gland, which can lead to clinical or subclinical mastitis; as a result, a reduction in milk production of > 20% is common (Keefe, 1997). In China, *S*. *agalactiae* infections have also been recorded in humans (Lee et al., 2021; Li et al., 2022), cattle (Hu et al., 2018; Lin et al., 2021; Han et al., 2022), and fish (He et al., 2020; Pu et al., 2020). In addition, *S*. *agalactiae* infections were reported in rabbits with acute respiratory distress syndrome, in the Sichuan Province (Ren et al., 2014), and in sheep with endometritis, in the Gansu Province of China (Han et al., 2020). However, research on the molecular epidemiology of *S*. *agalactiae* in dairy goats with mastitis has been scarcely documented in China. China has one of the largest dairy goat populations in the world: more than 1,290,000 dairy goats have been maintained in different-sized herds, including a large number of backyard farms (Luo et al., 2019). On these farms, most goats are fed by having a free range on grassland, sharing the same habitat with free-range cattle. Limited control measures have been adopted by these backyard farms; thus, the spread of endemic disease (Kuster et al., 2015; Hernandez-Jover et al., 2016; Pires et al., 2019) has been reported in many studies. Whether or not this contact affects the epidemics of *S*. *agalactiae* in dairy goats is unclear. In addition, poor milking hygiene is believed to accelerate the spread of mastitis (McDonald, 1984); specifically, the sharing of towels among goat milkers in backyard farms allows the transmission of *S*. *agalactiae via* milkers’ hands.

Many studies have investigated the variations of human and bovine *S*. *agalactiae*. Multilocus sequence typing (MLST) has been employed to identify strains, and is based on examining the allelic variations in seven slowly evolving housekeeping genes. Isolates are then classified into sequence types (STs), which can then be further clustered into clonal complexes (CCs) that are based on sequence similarities (Maiden et al., 1998). Our previous study showed that four CCs (i.e., CC64, CC67, CC103, and CC314) by MLST were identified in *S. agalactiae* strains isolated from dairy cattle in central and north-east China (Hu et al., 2018). In 2020, another study identified six CCs (i.e., CC4, CC23, CC64, CC67, CC103, and CC312) by MLST in dairy cattle in eastern, central, northern, and southern China. CC64, CC67, and CC103 were detected in both studies, but different CCs were also observed in the same region. Similarly, numerous CCs were also observed in *S. agalactiae* isolated from bovine herds in different Brazilian states (Carvalho-Castro et al., 2017). These studies indicate that genetic diversity between *S. agalactiae* in bovine is common. To our knowledge, there have not been any studies about the prevalent CCs of *S. agalactiae* strains from dairy goats in China; thus, molecular epidemiology data are not available.

The pathogenicity of *S. agalactiae* depends on multiple virulence factors, including neuraminidase and lipoteichoic acid, capsular polysaccharide antigen, pyrogenic exotoxin, M protein, the Christie–Atkins–Munch–Peterson (CAMP) factor, and hemolysin. These factors can increase the ability of *S. agalactiae* to invade and colonize its host (Oliveira et al., 2006; Emaneini et al., 2016). Furthermore, different virulence factors are indicated by different genes. For example, factors related to bacterial adhesion are encoded by *lmb*, *pavA*, *fbsA*, and *fbsB* (Gutekunst et al., 2004; Tenenbaum et al., 2005; Tenenbaum et al., 2007; Santi et al., 2009), whereas factors associated with immune evasion are encoded by *scpB*, *cspA*, *bac*, and *bca* (Beckmann et al., 2002; Harris et al., 2003; Beigverdi et al., 2014).

Currently, antimicrobial therapy is extensively adopted in the treatment of *S. agalactiae* infection in dairy herds and humans. However, the emergence of antibiotic-resistant *S. agalactiae* strains is continually found in the clinic; thus, antibiotics are becoming ineffective (Nagano et al., 2008; Kimura et al., 2008; Kimura et al., 2013). In addition, the increasing levels of antimicrobial residues in milk are a danger to public health because they cause adverse reactions in individuals who are allergic to antimicrobials (Tomazi et al., 2018). Furthermore, resistance genes within *S. agalactiae* can be transferred to antibiotic-susceptible bacteria, which can lead them to also become resistant to antibiotics (Mendes et al., 2019). In China, national antimicrobial resistance monitoring and surveillance programming in animals have taken place for many years. Nevertheless, knowledge of the antibiotic resistance of *S. agalactiae* from dairy goats remains lacking.

The aim of this study is to investigate the distribution of *S. agalactiae* isolates and to detect the presence of resistance and virulence genes in isolates from Chinese dairy goat farms in different regions from 2015 to 2021. The results of this study may serve as a data source of molecular epidemiology to control goat mastitis and guide the treatment regimen of dairy goats.

## Materials and methods

### Sample collection, bacterial isolation, and identification

All dairy goats in this work were raised free range in backyard farms, with a total of 20 to 50 goats per herd. Milking was performed, mainly by hand, twice per day. Pre- and post-milking teat disinfections were performed irregularly, and the cleaning agents, concentrations, mode of application, and duration of each disinfection and frequency of cleaning are shown in **Supplementary Table 1**. A total of 305 batches of raw milk were collected from dairy goats with clinical mastitis from 20 farms in 12 provinces in China from 2015 to 2021 (**Table 1**). Each milk sample was collected under aseptic conditions from goats, placed into sterile tubes, and stored in an ice box at 4°C for transportation to the laboratory for bacterial isolation.

**Table 1:** Goat milk samples with clinical mastitis collected from backyard goat dairy farms in five regions of China from 2015 to 2021.

Region	Province	Farm	Samples (*n*)	Samples with confirmed *S. agalactiae*	
				By herd, *n* (%)	By region (%)
North-east China	Heilongjiang	1	11	2 (18.2)	10.7
	Liaoning	2	13	2 (15.4)	
	Jilin	3	12	1 (8.3)	
	Inner Mongolia	4	19	3 (15.8)	
North-west China	Shanxi	5	22	2 (9.1)	13.6
		6	18	3 (16.7)	
		7	25	4 (16.0)	
	Gansu	8	16	2 (12.5)	
Central China	Henan	9	17	2 (11.8)	18.6
		10	22	4 (18.2)	
		11	14	3 (21.4)	
		12	10	2 (20.0)	
		13	13	2 (15.4)	
	Hubei	14	15	3 (20.0)	
		15	11	3 (27.3)	
Eastern China	Shandong	16	14	2 (14.3)	15.4
	Anhui	17	12	2 (16.7)	
Southern China	Guizhou	18	13	3 (23.1)	22
	Yunnan	19	11	2 (18.2)	
	Hunan	20	17	4 (23.5)	
Total			305	51 (16.7)	16.7

Milk samples were streaked on Columbia Blood Agar Base medium with 5% defibrinated sheep blood, and plates were incubated at 37°C for 24 h. Based on the characteristics of colony morphology, suspected colonies of *S. agalactiae* were subjected to Gram staining and confirmed as *S. agalactiae via* PCR by detecting the *dltS* gene (Poyart et al., 2007). Bacterial DNA was extracted using an EasyPure Bacteria Genomic DNA kit (TransGen Biotech, China), as per the manufacturer’s instructions. The extracted DNA was dissolved in 100 µl of double-distilled water, and the quantity and quality of DNA were measured using a spectrophotometer (UV1000, Techcomp, China). DNA samples were stored at –20°C for further downstream PCR analysis. One single *dltS* gene-positive isolate from each sampled goat was selected and stored at –70°C for further antibiotic susceptibility testing.

### Multilocus sequence typing (MLST)

As our previous work described (Hu et al., 2018), all 51 *S. agalactiae* isolates were typed using MLST by sequencing seven housekeeping genes (i.e., *adh*, *pheS*, *atr*, *glnA*, *sdhA*, *glcK*, and *tkt*) (Jones et al., 2003). Specific primers for these genes are available on the *S. agalactiae* MLST website (http://pubmlst.org/sagalactiae/). After performing PCR on each isolate, sequence types (STs) were assigned by analysis of the allele profile in the MLST database (http://pubmlst.org/sagalactiae/). Based on the eBURST algorithm program of Phyloviz (version 2.0a, www.phyloviz.net/), clonal complexes (CCs) were determined in all *S. agalactiae* strains.

### Antibiotic susceptibility testing

The standards for *S. agalactiae* disk diffusion methods proposed in the Clinical and Laboratory Standards Institute (CLSI)’s guidelines (CLSI, 2018) were used to determine the growth zone diameter. The inhibition zones were measured, recorded, and interpreted in accordance with CLSI guidelines (CLSI, 2018), and the instructions from antibiotic-sensitive papers (Hangzhou Microbial Reagent Company, China) were used as a reference when the antibiotics tested and interpreted were not available in the CLSI guidelines. Eighteen antibiotics were tested in the drug susceptibility test. Each goat *S. agalactiae* strain was tested five times to ensure reproducibility, and a *Streptococcus pneumoniae* strain (ATCC 49619) was used as a quality control strain.

### Detection of resistance and virulence genes

Bacterial genomic DNA was extracted as described in MLST. The presence of selected antibiotic resistance genes and virulence genes was detected by PCR analysis. Based on the class of antimicrobials used in the antibiotic susceptibility test and the most prevalent related genes revealed by previous studies in China (Tian et al., 2019; Han et al., 2022), the following resistance genes were detected: β-lactam resistance gene *pbp2b* (Ding et al., 2016); tetracycline resistance genes *tetL*, *tetK*, *tetM*, and *tetO* (Lopardo et al., 2003); macrolide resistance genes *ermA*, *ermB*, and *mefA* (Gao et al., 2012); aminoglycoside resistance genes *aphA3* and *aad6* (Poyart et al., 2003); lincosamide resistance gene *lnu(B)* (previously *linB*) (Bozdogan et al., 1999); and quinolone resistance genes *gyrA* and *parC* (Schmitt-Van de Leemput and Zadoks, 2007). All resistance gene primers are shown in **Table 2**. Twelve genes related to virulence based on those found in previous reports were screened by PCR and were as follows: *bac*, *bca*, *cfb*, *cspA*, *cylE*, *hylB*, *scpB*, *bibA*, *lmb*, *pavA*, *fbsA*, and *fbsB* (Shome et al., 2012; Kayansamruaj et al., 2014; Emaneini et al., 2016). All virulence gene primers are shown in **Table 3**.

**Table 2:** Primers of resistance genes.

Gene name	Sequence (5′–3′)	Product size (bp)	Annealing temperature (℃)	Reference
*Pbp2b*	F: GATCCTCTAAATGATTCTCAGGTGG	1,500	55	Ding et al. (2016)
	R: CCATTAGCTTAGCAATAGGTGTTGG			
*tetL*	F: TGGTGGAATGATAGCCCATT	229	50	Lopardo et al. (2003)
	R: CAGGAATGACAGCACGCTAA			
*tetM*	F: GGGGGGGGGGATGAAAATTATTAATATTGG	1,939	50	Lopardo et al. (2003)
	F: CCCCCCCCACTAAGTTATTTTATTGAACAT			
*tetK*	F: GATCAATTGTAGCTTTAGGTGAAGG	155	53	Lopardo et al. (2003)
	F: TTTTGTTGATTTACCAGGTACCATT			
*tetO*	F: GGGGGGGCACATGAAAATAATTAACTTAGG	1,936	51	Lopardo et al. (2003)
	R: GGGCGGTTAAGCTAACTTGTGGAACA			
*ermA*	F: TCTAAAAAGCATGTAAAAGAA	645	46.9	Gao et al. (2012)
	R: CTTCGATAGTTTATTAATATTAGT			
*ermB*	F: GCGGATCCATGAACAAAAATATAAAAT	751	50.0	Gao et al. (2012)
	R: GCGTCGACTTTCCTCCCGTTAAATAAT			
*mefA*	F: AGTATCATTAATCACTAGTGC	400	47.5	Gao et al. (2012)
	R: TTCTTCTGGTACTAAAAGTGG			
*AphA3*	F: TCTGCAGGTAAGTAAGTGCG	848	55	Poyart et al. (2003)
	R: GGGGTACCTTTAAATACTGTAG			
*aad-6*	F: TCTGGATCCTAAAACAATTCATCC	978	55	Poyart et al. (2003)
	R: CTGTAATCACTGTTCCCGCCT			
*lnu(B)*	F: CCTACCTATTGTTTGTGGAA	926	54	Bozdogan et al. (1999)
	R: ATAACGTTACTCTCCTATTC			
*gryA*	F: CGATGTCGGTCATTGTTG	496	50.5	Schmitt-Van de Leemput et al. (Schmitt-Van de Leemput and Zadoks, 2007)
	R: ACTTCCGTCAGGTTGTGC			
*parC*	F: CTGAATGCCAGCGCCAAAT	567	56	Schmitt-Van de Leemput et al. (Schmitt-Van de Leemput and Zadoks, 2007)
	R: GCGCATACGCACTGAACC			

F, forward; R, reverse.

**Table 3:** Primers of virulence genes.

Gene name	Sequence (5′–3′)	Product size (bp)	Annealing temperature (℃)	Reference
*bac*	F: CTATTTTTGATATTGACAATGCAA	592	58	Emaneini et al. (2016)
	R: GTCGTTACTTCCTTGAGATGTAAC			
*bca*	F: TAACAGTTATGATACTTCACAGAC	535	55	Emaneini et al. (2016)
	R: ACGACTTTCTTCCGTCCACTTAGG			
*cfb*	F: GCTGTTTGAAGTGCTGCTTG	288	60	Shome et al. (2012)
	R: GACTTCATTGCGTGCCAAC			
*cspA*	F: GGTCGCGATAGAGTTTCTTCCGC	104	58	Kayansamruaj et al. (2014)
	R: AACGCCTGGGGCTGATTTGGC			
*cylE*	F: TTCTCCTCCTGGCAAAGCCAGC	124	58	Kayansamruaj et al. (2014)
	R: CGCCTCCTCCGATGATGCTTG			
*hylB*	F: TCTAGTCGATATGGGGCGCGT	136	58	Kayansamruaj et al. (2014)
	R: ACCGTCAGCATAGAAGCCTTCAGC			
*scpB*	F: AGTTGCTTCTTACAGCCCAGA	567	58	Shome et al. (2012)
	R: GGCGCAGACATACTAGTTCCA			
*bibA*	F: AACCAGAAGCCAAGCCAGCAACC	127	58	Kayansamruaj et al. (2014)
	R: AGTGGACTTGCGGCTTCACCC			
*lmb*	F: AGTCAGCAAACCCCAAACAG	397	57	Shome et al. (2012)
	R: GCTTCCTCACCAGCTAAAACG			
*pavA*	F: TTCCCATGATTTCAACAACAAG	495	58	Shome et al. (2012)
	R: AACCTTTTGACCATGAATTGGTA			
*fbsA*	F: GTCACCTTGACTAGAGTGATTATT	85	58	Kayansamruaj et al. (2014)
	R: CCAAGTAGGTCAACTTATAGGGA			
*fbsB*	F: TCTGTCCAACAGCCGGCTCC	144	58	Kayansamruaj et al. (2014)
	R: TTCCGCAGTTGTTACACCGGC			

F, forward; R, reverse.

The PCR amplification reactions were carried out with an EasyTaq^®^ PCR SuperMix kit (TransGen, Beijing, China) in a total volume of 20 μl, containing 10 μl of 2 × EasyTaq^®^ PCR SuperMix, 0.4 μM of each primer, and 20 ng of template DNA. The amplification conditions were as follows: predenaturation at 94°C for 5 min, followed by 30 cycles of 30 s at 94°C, 30 s at an appropriate annealing temperature determined by the specific resistance and virulence gene primers, 30 s at 72°C, and a final extension at 72°C for 10 min. Samples with goat DNA or without genomic DNA were included as controls. The amplified products were electrophoresed on a 2% agarose gel in the presence of GelStain Blue (TransGen, Beijing, China) at 120 V for 60 min.

## Results

### Isolation and identification of *S. agalactiae*

After Gram staining and species-specific PCR to detect the *dltS* gene, 51 bacterial isolates in 305 milk samples were identified as *S. agalactiae*, with an isolation rate of 16.7%. The prevalence of the *S. agalactiae* infection rate in the five regions was 10.7% (8/75) in north-eastern China, 13.6% (11/85) in north-west China, 18.6% (19/102) in central China, 15.4% (4/26) in eastern China, and 22.0% (9/41) in southern China. The highest isolation rate in the provinces was 23.5% from Hunan Province in southern China, and the lowest isolation rate was 8.3% from Jilin Province in north-eastern China. The details of the *S. agalactiae* isolates are shown in **Table 1**. As shown in **Supplementary Table 1**, the cleaning agents, concentrations, mode of application, duration for each disinfection and the frequency of cleaning varied between farms. Some farms using the same disinfection practice showed similar isolation rates (farms 1, 6, 8, and 17), whereas some farms using the same disinfection practice showed notably different isolation rates (farms 3, 7, and 10). One farm (3) using a low concentration of cleaning agent showed a lower isolation rate than farms (1, 6, 8, 17) that used a higher concentration of cleaning agent; two farms (7, 10) using a low concentration showed similar isolation rates to farms (1, 6, 8, 17) using higher concentrations; and one farm (19) using a low concentration showed a lower isolation rate than that of a farm (5) using a higher concentration. Some farms (11, 12, 18) with a lower frequency (no more than once per day) of cleaning showed higher rates of isolation than farms with a higher frequency of cleaning (2, 15, 4). The farms (12, 15) using chlorhexidine with lower disinfection time and frequency showed high isolation rates.

### MLST analysis

**Figure 1:**
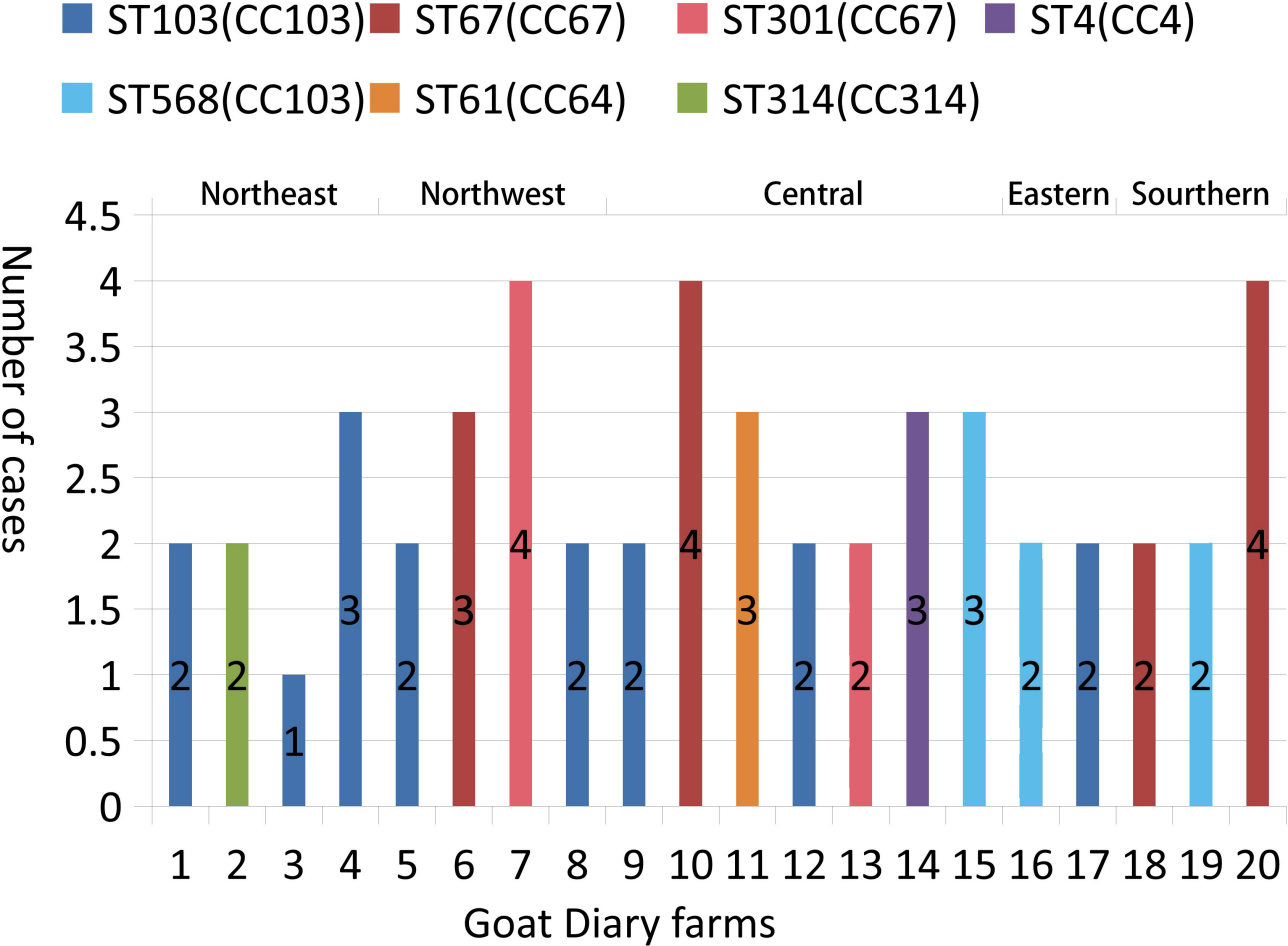
Typing of 51 *S. agalactiae* strains present in 20 goat dairy farms in China. The numbers of *S. agalactiae* are shown in columns. ST indicates the MLST sequence type; CC indicates the clonal complexes.

The results of the MLST analysis are shown in **Figure 1**. Among the 51 *S. agalactiae* strains, seven unique STs (ST-4, ST-61, ST-67, ST-103, ST301, ST-314, and ST-568) and five CCs (CC4, CC64, CC67, CC103, and CC314) were identified. The largest segments of the strains were ST-103 (*n* = 16) and ST568 (*n* = 7), which were both clustered into CC103 and had been detected in five regions, including 10 provinces (farms 1, 3, 4, 5, 8, 9, 12, 15,16, 17, and 19). The second largest segments of the strains were ST-67 (*n* = 14) and ST-301 (*n* = 6), which were both clustered into CC67 and were detected in three regions, including four provinces (farms 6, 7, 10, 13, 18, and 20). ST-4 (*n* = 3), grouped into CC4, was detected in the Hubei Province only. ST61 (*n* = 3), grouped into CC64, was detected in the Henan Province only. ST-314 (*n* = 2), grouped into CC314, was detected in the Liaoning Province only. Based on the ST described, within the same farm only one ST was observed; however, within the same province (Shanxi, Henan, and Hubei), different STs could be observed on different farms.

### Antimicrobial susceptibility and resistance genes

The 51 *S. agalactiae* strains isolated from milk samples from goats with clinical mastitis were categorized as susceptible, intermediate, or resistant to 18 antibiotics (six classes). As shown in **Table 4**, isolates had different degrees of resistance to different antimicrobial agents, and the drug resistance rates from the highest to the lowest resistance were as follows: penicillin (100.0%), oxacillin (100.0%), amoxicillin (100.0%), lincomycin (86.3%), doxycycline (82.4%), tetracycline (76.5%), amikacin (74.5%), cefalotin (68.6%), ceftiofor (62.7%), spectinomycin (62.7%), kanamycin (58.8%), clindamycin (52.9%), gentamicin (51.0%), enrofloxacin (51.0%), erythromycin (49.0%), levofloxacin (41.2%), azithromycin (31.4%), and ciprofloxacin (29.4%). All isolates (100%) were multiresistant to three or more antimicrobial agents, and three isolates (5.9%) showed resistance to all the antimicrobial agents tested.

**Table 4:** The antimicrobials disk breakpoints and the distributions of antimicrobial resistance of 51 *S. agalactiae* strains isolated from milk samples of goats with clinical mastitis.

Antimicrobial class	Antimicrobial agent	Concentration (μg/piece)	Diameter of the bacteriostatic sphere			Isolates, *n* (%)		
			Susceptible	Intermediate	Resistant	Susceptible	Intermediate	Resistant
β-Lactams	Penicillin	10U	≥ 29	:	≤ 28	0 (0)	0 (0)	51 (100.0)
	Oxacillin	1	≥ 13	:	≤ 10	0 (0)	0 (0)	51 (100.0)
	Amoxicillin	10	≥ 18	:	≤ 13	18 (35.3)	7 (13.7)	26 (100.0)
	Ceftiofor	30	≥ 24	:	≤ 14	16 (31.4)	3 (5.9)	32 (62.7)
	Cefalotin	30	≥ 24	:	≤ 14	12 (23.5)	4 (7.8)	35 (68.6)
Tetracyclines	Tetracycline	30	≥ 15	:	≤ 11	10 (19.6)	2 (3.9)	39 (76.5)
	Doxycycline	30	≥ 16	:	≤ 12	9 (17.6)	0 (0)	42 (82.4)
Macrolides	Erythromycin	15	≥ 21	:	≤ 15	21 (41.2)	5 (9.8)	25 (49.0)
	Azithromycin	15	≥ 18	:	≤ 13	32 (62.7)	3 (5.9)	16 (31.4)
Aminoglycosides	Gentamicin	10	≥ 15	:	≤ 12	20 (39.2)	5 (9.8)	26 (51.0)
	Amikacin	30	≥ 17	:	≤ 14	13 (25.5)	0 (0)	38 (74.5)
	Kanamycin	30	≥ 18	:	≤ 13	20 (39.2)	1 (2.0)	30 (58.8)
	Spectinomycin	100	≥ 18	:	≤ 14	13 (25.5)	6 (11.8)	32 (62.7)
Lincosamides	Lincomycin	2	≥ 21	:	≤ 14	6 (11.8)	1 (2.0)	44 (86.3)
	Clindamycin	2	≥ 21	:	≤ 14	16 (31.4)	8 (15.7)	27 (52.9)
Quinolones	Levofloxacin	5	≥ 21	:	≤ 15	20 (39.2)	10 (19.6)	21 (41.2)
	Enrofloxacin	5	≥ 19	:	≤ 15	19 (37.3)	6 (11.8)	26 (51.0)
	Ciprofloxacin	5	≥ 21	:	≤ 15	29 (56.9)	7 (13.7)	15 (29.4)

The criteria applied for interpreting the zone diameter (mm) of drugs were in accordance with CLSI guidelines. If the antibiotics tested were not covered by the guidelines, the instructions for the use of antibiotic-sensitive papers (Hangzhou Microbial Reagent Company, China) were followed.

From a geographical perspective, the average percentages of resistant strains for six classes of antimicrobials in five regions of China are shown in **Table 5**. We found a clear distinction: the average percentage of resistant strains was much lower in southern China and east China than in the other three regions, and the isolates from the five regions of China were generally more resistant to β-lactams and tetracyclines, while also being more sensitive to macrolides and quinolones. (**Table 5**).

**Table 5:** The average rates of antimicrobial resistance of *S. agalactiae* isolated from goat milk samples in five regions of China.

Antimicrobial class	Average rates of antimicrobial resistance (%)				
	North-east	North-west	Central	East	South
β-Lactams	87.5	89.1	91.6	85.0	75.6
Tetracyclines	81.3	86.4	81.6	87.5	61.1
Macrolides	43.8	50.0	42.1	37.5	22.2
Aminoglycosides	65.6	63.6	56.6	50.0	72.2
Lincosamides	62.5	72.7	86.8	25.0	55.5
Quinolones	45.8	39.4	40.4	41.7	37.0
Average	67.4	68.7	68.1	55.6	57.4

**Figure 2:**
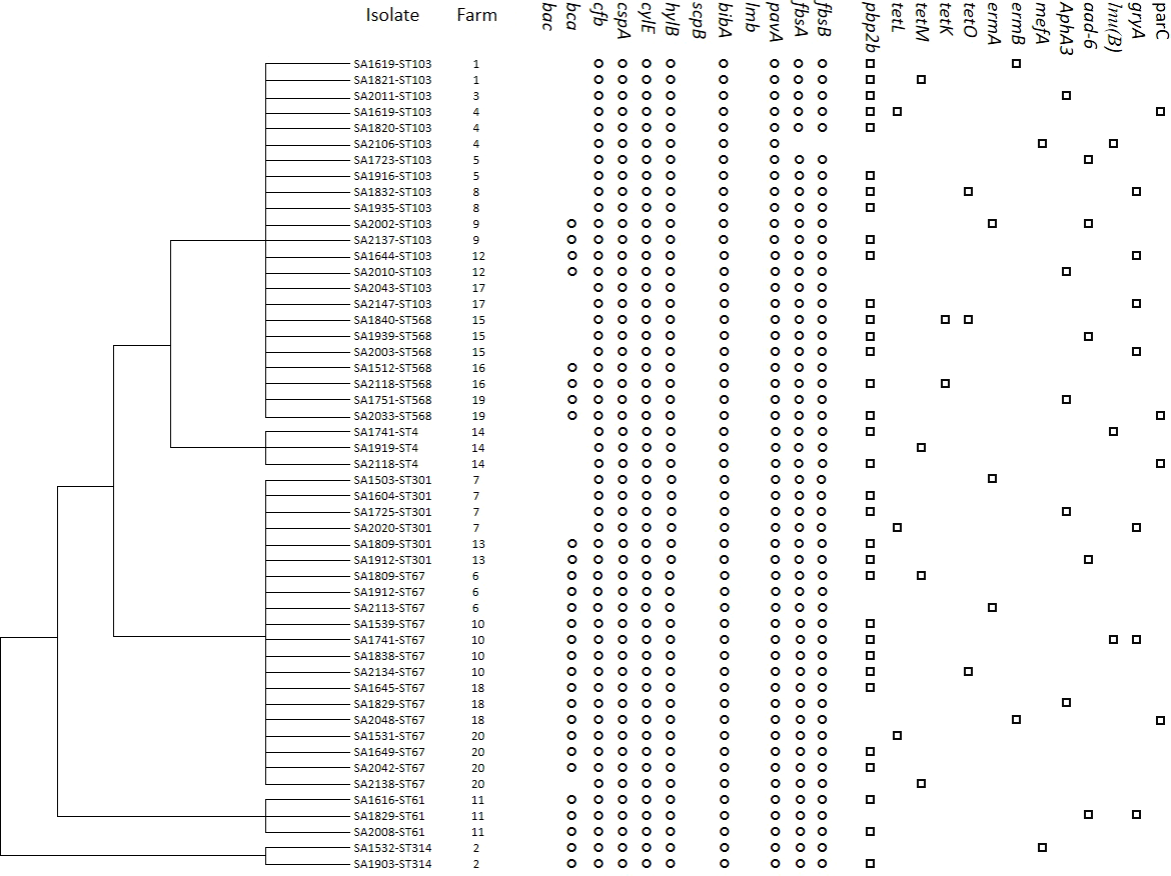
Cluster analysis of *S. agalactiae* isolated from goat with mastitis in China based on virulence-associated gene profiles. The presence (black) or absence (white) of genes, isolate name, goat farm, and gene names are shown. The virulence and antimicrobial resistance profiles are indicated on the right.

To investigate genetic antimicrobial resistance, 13 genes accounting for resistance to six antibiotic classes were screened by PCR, and the details of the results are shown in **Figure 2**. The detection rates of six classes of antimicrobial-related genes were also calculated. In total, 33 (64.7%) isolates carried β-lactam-related resistance genes (*pbp2b*) and could be observed in all five CCs in this work; 12 (23.5%) for tetracyclines (*tetL*, *tetM, tetK*, and *tetO*) in CC103, CC4, and CC67; 11 (21.6%) for quinolone-related resistance genes (*gryA*, *parC*) in CC103, CC4, CC67, and CC61; 10 (19.6%) for aminoglycosides (*aphA3* and *aad6*) in CC103, CC67, and CC61; seven (13.7%) for macrolides (*ermA*, *ermB*, and *mefA*) in CC103, CC67, and CC314; and three (5.9%) for lincosamide (*lnu(B)*) in CC103, CC67, and CC4. In summary, only three (5.9%) isolates (SA2043-ST103, SA1512-ST568, and SA1912-ST67) in CC103 and CC67 did not harbor a resistance gene; 25 (49.0%) in all five CCs harbored one resistance gene; 19 (37.3%) in CC103, CC4, CC67, and CC61 harbored two resistance genes; and four (7.8%) in CC103 and CC67 harbored three resistance genes. Genetic diversity in resistance genes was mostly observed in CC103 and was followed by CC67.

### Detection of virulence genes

Screening for the 12 *S. agalactiae* genes involved in virulence is shown in **Figure 2**, and a dendrogram was created based on virulence genes by MEGA 6.0. Moreover, the STs, farms, and resistance gene information were included. Three virulence genes, *bac*, *scpB*, and *lmb*, could not be detected in any of the 51 isolates, whereas *cfb*, *cspA*, *cylE*, *hylB*, *bibA*, and *pavA* were present in all the isolates. Only one isolate on farm 4 was negative for *fbsA* and *fbsB* (98.0%), and the frequency was lower for *bca* (51.0%). Considering the combinations of the virulence genes detected in each isolate, we observed three virulence gene profiles: profile 1 (*bca cfb*-*cspA*-*cylE-hylB-bibA-pavA-fbsA-fbsB*) was the most common profile, with a rate of 54.9%, followed by profile 2 (*cfb*-*cspA*-*cylE-hylB-bibA-pavA-fbsA-fbsB*), with a rate of 43.1%, and profile 3 (*cfb*-*cspA*-*cylE-hylB-bibA-pavA*), with a rate of 2.0%. On farms 4 and 20, two profiles were found on a single farm, and, on the remaining 18 farms, only one profile was found on each farm. Isolates in profile 1 belonged to six STs—ST314 (CC314), ST67 (CC67), ST103 (CC103), ST61 (CC64), ST301 (CC67), and ST568 (CC103)—on 11 farms in seven provinces; isolates in profile 2 belonged to four STs —ST103 (CC103), ST301 (CC67), ST4 (CC4), and ST568 (CC103) —on 10 farms in eight provinces; and a single strain in profile 3 belonged to CC103 on farm 4 in Inner Mongolia, which was unique.

## Discussion

In the present study, we isolated a total of 51 *S. agalactiae* strains in 305 milk samples from dairy goats with clinical mastitis on 20 farms from five regions of China between 2015 and 2021. The total isolation rate in this study was 16.7%, which was higher than that found in goats in Nigeria (11.0%) (Danmallam and Pimenov, 2019), in cows in Portugal (13.5%) (Rato et al., 2013) and Argentina (11.0%) (Hernandez et al., 2021), and in several studies from China, which reported rates of 8.71% (Tian et al., 2019), 11.1% (Lin et al., 2021), and 16.5% (Yang et al., 2013). In this work, we found that the prevalence of *S. agalactiae* was higher in central and southern China than in the other three regions; it is likely that the high average temperatures in those two regions accelerated the spread of *S. agalactiae* (Keefe, 1997). As shown in **Supplementary Table 1**, in this study the cleaning agents, concentrations, mode of application, and duration of disinfection, and the frequency of cleaning, contributed to the different isolation rates of different farms. The isolation rates were low in most farms using povidone iodine or sodium hypochlorite, and low rates were also associated with a longer disinfection time and higher frequency of cleaning (Jorgensen et al., 2016); however, a few exceptions were also observed. Furthermore, this result did not account for other factors, such as the hygiene status of farms, the application of disinfection on the farm, or the risk of exposure to cattle with mastitis when grazing (Lianou et al., 2020). In addition, the small number of samples collected in partial regions (eastern China and southern China) limited effective evaluation, and more sampling should be performed in the future. *S. agalactiae* is one of the major pathogens that causes mastitis, and the present study is the first to characterize *S. agalactiae* isolates circulating among dairy goats with clinical mastitis in China on a molecular level, further demonstrating that *S. agalactiae* is an important pathogenic factor of mastitis in goats and that more effective management to control *S. agalactiae* mastitis is imperative.

The MLST analysis revealed distinct heterogeneity among the 51 *S. agalactiae* strains, which were divided into seven STs and five CCs. CC103 and CC67 were the predominant CCs in the goat strains, while all the STs identified in this study have been previously reported in bovine isolates (Hu et al., 2018; Lin et al., 2021; Liu et al., 2022). Recently, the prevalence rate of CC103 in cattle in China was reported as 73.8% (Lin et al., 2021) or 97.9% (Liu et al., 2022), which is higher than that for CC103 in goats in this study (45.1%). On the other hand, this study revealed that the prevalence rate of CC67 (39.2%) was higher than reported in two previous investigations (9.5% and 0.0%). These results suggest that the hosts play a role in the epidemic strain group of *S. agalactiae*. CC67 is the most common CC among bovine isolates (Bisharat et al., 2004) and is transmitted *via* a contagious route (Jorgensen et al., 2016); therefore, when milkers in dairy goat backyard farms shared the same towels, unclean milkers’ hands accelerated the spread of the CC67 group. In addition, CC103 is an environmental pathogen (Cobo-Ángel et al., 2018) and has been reported in cattle in Asia, Europe, and South America (Brochet et al., 2006; Zadoks et al., 2011; Hu et al., 2018); therefore, it is unsurprising that CC103 is widespread on these goat farms. Furthermore, an investigation of the two main prevalent CC groups revealed that there are two transmission routes—goat to goat and environmental reservoir to goat—within herds. CC4, CC64, and CC314 have been found in goats and were also isolated from cattle in China (Hu et al., 2018; Lin et al., 2021). This distribution of CCs in goats in China is partially attributed to national and international animal trade. Meanwhile, we found only one ST in each herd, demonstrating homogeneity among *S. agalactiae* isolates. The highly infectious characteristics of *S. agalactiae* may very likely be the cause of this phenomenon, further indicating the same source of transmission between goats on the same farm (Rato et al., 2013). Similarly, strains that belong to the same cluster were also observed on cattle farms in China (Lin et al., 2021). Furthermore, to reduce the risk of spreading *S. agalactiae* to healthy goats, it is imperative to control the transmission from infected goats to healthy goats.

In China, commercial vaccines against *S. agalactiae* strains that cause mastitis are not available, so the main method of controlling mastitis in dairy goats is antimicrobial therapy; as a result, bacterial resistance to antimicrobial agents has been increasing year on year (Lin et al., 2021; Liu et al., 2022). To obtain a precise and deep insight into antimicrobial susceptibility, all isolates were tested against six antimicrobial classes, including 18 agents used for mastitis treatment in animals and/or in human medicine. The high β-lactam resistance rate found among *S. agalactiae* in this study is in accordance with previous work that focused on bovine *S. agalactiae* in Inner Mongolia (Ding et al., 2016), and in Heilongjiang, Liaoning, and Henan Provinces (Hu et al., 2018). Given that β-lactams have been the most commonly used antimicrobial classes for the treatment of mastitis, selective pressure in goat backyard farms has hastened the development of drug resistance. Similarly, in five regions, the isolates showed high resistance to tetracyclines, in the range of 61.1%–87.5%. High rates of resistance have also been observed in bovine *S. agalactiae* in China (Lin et al., 2021; Han et al., 2022; Liu et al., 2022) and in Brazil (Tomazi et al., 2018). Although tetracyclines are not the first-line agent in mastitis treatment, in recent years they have been one of the most commonly used antibiotics in animals worldwide according to the World Organisation for Animal Health (Health O-WOfA, 2018). This suggests the possibility that an increase in tetracycline resistance in *S. agalactiae* is a side effect of treatments for other bacterial infections. The isolate rates of resistance to the remaining four antimicrobial classes were lower than resistance to β-lactams and tetracyclines. Furthermore, all isolates were multiresistant; this phenomenon was also observed in bovine *S. agalactiae* in China (Tian et al., 2019). In addition, all streptococcal isolates in Denmark (Chehabi et al., 2019) and in the Emilia Romagna region in Italy (Carra et al., 2021) were found to be susceptible to β-lactam antibiotics. In our study, different antimicrobial resistance profiles were observed in different regions. The inconsistency between these reports and our present findings may be due to the different treatment regimens of these farms. In particular, irregular drug usage in clinics is a major driver of antimicrobial resistance (Barkus and Lisauskienė, 2016). This information indicates that antimicrobials should be used with discretion for the treatment of goat *S. agalactiae* mastitis unless a sensitive drug is selected by tests. To date, investigations on antimicrobial use in goat dairy herds in China are scarce; hence, data on the changes and trends in antimicrobial resistance over the past years are unavailable, and more detailed data on this topic would be useful for developing strategies to improve clinical treatment. Based on the results, macrolides and quinolones may be used in the future to treat mastitis caused by *S. agalactiae.* Meanwhile, reducing drug usage in dairy goats would lead to a reduction in health threats to humans, such as allergies and drug resistance (Hendriksen et al., 2008).

As one of the target enzymes for β-lactams, the presence of the *pbp2b* resistance gene was determined for all *S. agalactiae* isolates, of which 64.7% were positive. To date, most *S. agalactiae* isolates with the *pbp2b* gene have been isolated from human hosts (Nagano et al., 2008; Kimura et al., 2008), and only limited data are available on bovine strains (Hu et al., 2018). Our work is the first to show evidence of the *pbp2b* gene in goat isolates in China. In *S. agalactiae* isolates, tetracycline resistance is mediated by ribosome protection genes (i.e., *tetM* and *tetO*) or by efflux pump genes (i.e., *tetK* and *tetL*) (Rubio-López et al., 2012). Aminoglycoside resistance is mediated by genes encoding an aminoglycoside phosphotransferase. Macrolide resistance is mediated by a ribosome methylase encoded by the *ermA* or *ermB* genes, and an active efflux pump encoded by the *mef* gene (Ko et al., 2004). Lincosamide resistance is mediated by the *lnu(B)* gene encoding a lincosamide-inactivating nucleotidyltransferase. Isolates in this work were shown to be positive for these four antimicrobial class resistance genes, with rates of 23.5%, 19.6%, 13.7%, and 5.9%, respectively. These positive genes were also detected in bovine strains in China and Argentina (Tian et al., 2019; Hernandez et al., 2021). Quinolone resistance is mediated by genes encoding the type II topoisomerase enzymes DNA gyrase (*gyrA*) and topoisomerase IV (*parC*) (Simoni et al., 2018), and in this study 21.6% of isolates were positive. Similarly, these positive genes were also detected in bovine strains in China (Tian et al., 2019). As CC103 was a predominant CC group on the goat farms, genetic diversity for resistance was mostly observed in CC103. In this work, a discrepancy in the antibiogram profiling was observed: every isolate was resistant to at least three antibiotics, yet some of the isolated strains did not possess any of the antibiotic resistance genes that were tested. Screening only some resistance genes may be the cause for this discrepancy. Therefore, a whole-genome sequencing approach in future work would be a more effective way to obtain an antimicrobial resistance profile of these isolates without biasing the results.

In this study, the results of virulence gene detection showed that *cfb*, *cspA*, *cylE*, *hylB*, *bibA*, and *pavA* were present in all the isolates, whereas the *bac*, *scpB*, and *lmb* genes were not. The detection rates of all genes, with the exception of the *pavA* gene, were the same as those reported for bovine isolates, including ST4, ST23, ST67, ST103, ST312, ST568, and ST 570, in China (Lin et al., 2021). In Brazilian bovine isolates, the *cfb* and *hylB* genes appear to be the most prevalent (Carvalho-Castro et al., 2017). Our work first identified these virulence genes (ST61, ST301, and ST314 groups) in strains isolated from dairy animals. In addition, the *fbsA*, *fbsB*, and *bca* genes were detected in more than half of the isolates. These virulence factors play roles in the adhesion to and invasion of host cells (Gao et al., 2012). For example, the *cfb* gene encodes the hemolysis-promoting factor CAMP, which can activate Fab fragments of immunoglobulin and then decrease the immune response (Lasagno et al., 2011). It is considered to be one of the major etiological factors of *Streptococcus* infection (Wu et al., 2016). *cylE* is a toxin involved in tissue damage and dissemination of *S. agalactiae* in the host (Reiss et al., 2011). *bibA* is related to the expression of bacterial immunogenic adhesin (Santi et al., 2009). Further work involving toxicological tests would shed light on the roles of these virulence factors. The *lmb* gene is related to the adherence of *S. agalactiae* and has been found in isolates from humans; however, many studies have indicated the lack of this gene in bovine strains (Lin et al., 2021; Han et al., 2022). In line with these findings, we detected no *lmb* gene in any goat isolates work. Negative results for the virulence genes *bac* and *scpB* were also observed in bovine isolates in Argentina (Hernandez et al., 2021).

## Conclusions

Fifty-one *S. agalactiae* strains isolated from 305 milk samples collected from goats with mastitis in 13 provinces of China were investigated in this study. A total of 18 antibiotics in 6 classes were tested. There were 7 different STs in 5 CCs were identified according to MLST; CC-103 and CC67 strains were predominant, and all of these STs were first identified in dairy goat farms in China. Meanwhile, 13 genes accounting for resistance to 6 antibiotic classes and 9 genes associated with virulence were first identified in goat isolates. This work provides a primary source for the molecular epidemiology of *S. agalactiae* in dairy goat herds in China. Furthermore, there is an urgent need for a national strategy to strengthen the reasonable utilization of antimicrobials by veterinarians and herd farms. Owing to the limited sample size, further investigation is necessary to confirm the current results and determine how *S. agalactiae* can be best controlled in dairy goats.

## Data availability statement

The original contributions presented in the study are included in the article/**Supplementary material**. Further inquiries can be directed to the corresponding authors.

## Ethics statement

The animal study was reviewed and approved by the Animal Welfare and Ethics Committee of Nanyang Normal University. Written informed consent was obtained from the owners for the participation of their animals in this study.

## Author contributions

HS participated in sample collection, pathogen isolation, and participated in the design of the study. MZ participated in MLST, antibiotic resistance testing, and drafting the main parts of the manuscript. ZZ, YH, and SS participated in antibiotic resistance gene detection testing. RH, LW, and GL participated in virulence gene detection. LY participated in the design of the study and revised the manuscript. All authors read and approved the final manuscript.
