# Supplementary material for: Molecular epidemiology, drug resistance, and virulence gene analysis of Streptococcus agalactiae isolates from dairy goats in backyard farms in China
Source: Front Cell Infect Microbiol. 2023 Jan 9;12:1049167. doi: 10.3389/fcimb.2022.1049167 (PMC9868259; doi:10.3389/fcimb.2022.1049167)
Supplement: Supplementary file 1 [file Table_1.docx]

**Supplementary table 1** The milking disinfection practices in 20 farms

| Farm | Cleaning agents | Concentrations | Mode of application ^a^ | Time duration for each disinfection | Frequency of cleaning ^b^ |
| --- | --- | --- | --- | --- | --- |
| 1 | povidone iodine | 1% | Pre/post | 30s | 4/d |
| 2 | [sodium hypochlorite](javascript:;) | 3% | Pre/post | 30s | 2/d |
| 3 | povidone iodine | 0.5% | Pre/post | 30s | 4/d |
| 4 | [potassium permanganate](javascript:;) | 0.02% | Pre/post | 20s | 4/d |
| 5 | [sodium hypochlorite](javascript:;) | 4% | Pre/post | 30s | 4/d |
| 6 | povidone iodine | 1% | Pre/post | 30s | 4/d |
| 7 | povidone iodine | 0.5% | Pre/post | 30s | 4/d |
| 8 | povidone iodine | 1% | Pre/post | 30s | 4/d |
| 9 | povidone iodine | 1% | Pre/post | 30s | 2/d |
| 10 | povidone iodine | 0.5% | Pre/post | 30s | 4/d |
| 11 | [sodium hypochlorite](javascript:;) | 3% | post | 30s | 1/d |
| 12 | [chlorhexidine](javascript:;) | 0.5% | post | 30s | <1/d |
| 13 | povidone iodine | 1% | Pre/post | 20s | 4/d |
| 14 | povidone iodine | 1% | post | 20s | 2/d |
| 15 | [chlorhexidine](javascript:;) | 0.5% | pre | 20s | 2/d |
| 16 | povidone iodine | 1% | Pre/post | 20s | 4/d |
| 17 | povidone iodine | 1% | Pre/post | 30s | 4/d |
| 18 | [potassium permanganate](javascript:;) | 0.02% | pre | 30s | 1/d |
| 19 | [sodium hypochlorite](javascript:;) | 3% | Pre/post | 30s | 4/d |
| 20 | povidone iodine | 0.5% | pre | 20s | 2/d |

^a^: pre: pre-milking teat disinfection; post: post-milking teat disinfection; Pre/post: pre-milking teat disinfection and post-milking teat disinfection.

^b^: 1/d:once per day; <1/d: less than once per day; 2/d: twice per day; 4/d: four times per day.
